# Supplementary material for: Exploring perceived walkability in one-way commercial streets: An application of 360° immersive videos
Source: PLoS One. 2024 Dec 30;19(12):e0315828. doi: 10.1371/journal.pone.0315828 (PMC11684695; doi:10.1371/journal.pone.0315828)
Supplement: S1 Questionnaire — (DOCX) [file pone.0315828.s001.docx]

# **S1 Appendix**

**Questionnaire**

Reponses to the following questions were recorded using Google forms

**Preliminary questionnaire**

- Year of birth, gender (male/female)
- Do you wear glasses? (Yes/No)
- Do you have any health problem? (vision, hearing, others)
- Do you have driving license? (Yes/No)
- Which transportation mode do you use most? ( car/bicycle/motorcycle/ public transport/ others)
- Have you ever had a car accident experience? (Yes/No).
- Which kinds of accident? (car-related/ motorbike-related/ bike-related/ walking)
- Do you have any experience using VR? (Yes/No)

Perception of safe walking (strongly disagree/ disagree/ neutral/ agree/ strongly agree)

- When I'm in a hurry, I cross the street without using the crosswalk.
- Even if I'm not in a hurry, I jaywalk.
- If there are no passing vehicles, I jaywalk.
- I jaywalk if there is a destination across the street.
- Compared to other people, I tend to be more careful when crossing the road.
- I walk on the roadway even when there is a sidewalk.
- When cars are stopped on a busy road, I cross the street while avoiding them.
- I cross without paying attention to the flow of traffic.
- I get distracted while walking.
- I use my cellular phone when crossing.
- I cross the street even if I cannot clearly see my surroundings due to obstacles.
- I am less likely to be involved in a car accident than other people
- Considering the risk of traffic accidents, I am willing to take a detour
- I am willing to take risks to save time

**Experiment questionnaire**

All of the items of the perceived walkability questionnaire are answered on a 5-point Likert scale after completing each scenario. The scale ranges from 1 (strongly disagree) to 5 (strongly agree)

- This street is safe for walking.
- This street is safe for crossing
- This street is convenient for walking
- This street is attractive for walking
- This street is the street I want to walk.

**Following-up questionnaire**

- Do you think the differences between each scenario are clear? (strongly disagree/ disagree/ neutral/ agree/ strongly agree)
- Do you think the differences in street environment of each scenario affect transportation safety? (strongly disagree/ disagree/ neutral/ agree/ strongly agree)What street environment factors influence pedestrian traffic safety? (one-way street, one-way vehicle approach direction, presence of sidewalk, road pavement, building along street, street furniture, parked car, other(open field))

VR experiment immersion (strongly disagree/ disagree/ neutral/ agree/ strongly agree)

- I felt like the presented situation was real.
- I felt like I had been to the presented place.
- I immersed myself in the presented situation and conducted the experiment.
- The experience through the experiment was intense.

VR experiment sickness (strongly disagree/ disagree/ neutral/ agree/ strongly agree)

- I was dizzy during the experiment.
- I felt nauseous during the experiment.
- I got a headache during the experiment.
- I felt eye strain during the experiment.
