# Supplementary material for: Exploring perceived walkability in one-way commercial streets: An application of 360° immersive videos
Source: PLoS One. 2024 Dec 30;19(12):e0315828. doi: 10.1371/journal.pone.0315828 (PMC11684695; doi:10.1371/journal.pone.0315828)
Supplement: Attachment — Submitted filename: Response to reviewers_final.docx [file pone.0315828.s003.docx]

**Response to Reviewer’s Comments and Suggestions**

We appreciate the careful remarks and comments from the reviewers. Based on their feedback, we have made substantial revisions, including reorganizing the literature review section and clarifying the explanations of the results. Our responses to the reviewers’ feedback are detailed under each comment.

**Editor**

**Journal requirements:**

**When submitting your revision, we need you to address these additional requirements.**

**1. Please ensure that your manuscript meets PLOS ONE's style requirements, including those for file naming. The PLOS ONE style templates can be found at**
[**https://journals.plos.org/plosone/s/file?id=wjVg/PLOSOne_formatting_sample_main_body.pdf**](https://journals.plos.org/plosone/s/file?id=wjVg/PLOSOne_formatting_sample_main_body.pdf) **and**
[**https://journals.plos.org/plosone/s/file?id=ba62/PLOSOne_formatting_sample_title_authors_affiliations.pdf**](https://journals.plos.org/plosone/s/file?id=ba62/PLOSOne_formatting_sample_title_authors_affiliations.pdf)

- We have followed the style requirements in writing the manuscript and presenting the tables, figures, and references in the revised manuscript.

**2. Thank you for stating the following financial disclosure: 
 [This research was supported by the National Research Foundation of Korea (NRF: 2021R1A2C2011106).].  
Please state what role the funders took in the study.  If the funders had no role, please state: "****The funders had no role in study design, data collection and analysis, decision to publish, or preparation of the manuscript." 
If this statement is not correct you must amend it as needed. 
Please include this amended Role of Funder statement in your cover letter.**

- In the Acknowledgement, we added the statement of the role of funder: "The funders had no role in study design, data collection and analysis, decision to publish, or preparation of the manuscript."

**Additional Editor Comments (if provided):

[Note: HTML markup is below. Please do not edit.]**

**Reviewers' comments:

Reviewer's Responses to Questions**

**Comments to the Author**

**Please use the space provided to explain your answers to the questions above. You may also include additional comments for the author, including concerns about dual publication, research ethics, or publication ethics. (Please upload your review as an attachment if it exceeds 20,000 characters)**

- To make reviewers easily follow our revision, we tried to explain your answers, which the information of line number is based on the track change version.

**Reviewer #1:**

1. **Add Separate section on objectives of study with clearly stated objectives along with limitations of the study.**

- To clearly state the objectives and limitations of the paper, we have separated the literature review from the introduction. In the introduction section, we focus on presenting the background and objectives of this study while discussing its primary limitation related to the scope of the research. Additionally, suggestions for addressing the limitations of this study in future research are provided in the conclusion section.
- Introduction: We presented the objectives, research questions, and a primary limitation of this study (see Lines 81-108).
- Conclusion: We also provided suggestions for how the limitations of this study can be addressed in future research (see Lines 573-584).

2. **Conclusion should be separated by data. No references in conclusion section.**

- As suggested, we divided the conclusion and discussion. In this fashion, there were no references in the conclusion section. (See Line 555-584).

3. **Add labels to x and Y axis in the figures along with legends.**

- As suggested, we added labels to the x-axis and y-axis in Figures 3, 4, and 5, along with legends (see Figures 3, 4-1, 4-2, 5).

**Reviewer #2:**

**Manuscript PONE-D-24-28229 explores the perceived walkability of one-way commercial streets by utilizing immersive 360-degree virtual reality (VR) videos. Although there are many studies on walkability, this study is unique in its use of VR. As a result, the presence of sidewalks is considered critical factor contributing to enhanced perceived walkability on narrow commercial street. The findings of manuscript PONE-D-24-28229 could be utilized for more systematic walkability assessments and urban design improvements, especially in constrained road spaces.
The reviewer evaluated the manuscript as revision. That is because the reviewer has some issues with your manuscript.**

**Introduction**
**Ln 94-95**
**Employing an immersive experimental environment can enhance our understanding of how street design influences subjective walkability.
The question here concerns the originality of your research. Nevertheless, you have not answered this question in your Discussion section. Walkability has been studied in a variety of ways, including questionnaires, GIS, etc. In contrast, did the methods used in your VR have the same results? Were they different results?**

- We appreciate your comment. A unique and significant contribution of this study is the application of VR to evaluate perceived walkability in narrow one-way commercial streets. As suggested, we presented the advantages of using VR to investigate the multifaceted aspects of perceived walkability in depth in the discussion section. Specifically, VR can overcome biased recall evaluations and generate fine-grained subjective data at the micro-scale level, which cannot be achieved through traditional on-site or recall-based surveys. (see Lines 514-523)
- We also emphasized the contribution of this study in the conclusion, highlighting the application of VR. (see Lines 557-561)
- As mentioned in the introduction of this study, the walkability of one-way streets is a contentious topic, making it generally difficult to describe the results of this study as similar to or different from previous research. In this study, when the results of analyzing perceived walkability in one-way commercial streets using VR aligned with previous research, we cited those studies to demonstrate consistency with existing literature. Below are a couple of examples.
- Line 422 / Line 509 / Line 530

**Material and Methods
Ln 111
a mean age of 26.95 and 111 an age range of 21–39
You need to describe in detail the socio-demographics of the participants. In your study, the questionnaire asked about personal information, such as demographics, health status, driving experience, collision and traffic accident history, habits of walking on commercial streets, familiarity with virtual environments, and other relevant details. You must clearly state in your manuscript whether these sociodemographic characteristics influenced the results.**

- As suggested, we have included a detailed description of the socio-demographics of the participants in the Results section. This includes demographics, health status, driving experience, collision and traffic accident history, habits of walking on commercial streets, and familiarity with virtual environments.

*“The study recruited 40 undergraduate, graduate, and research students from Ulsan National Institute of Science and Technology..."* (see Lines 326-336).

- Overall, we utilized these sociodemographic characteristics as control variables in the regression model to minimize bias. Additionally, a random intercept for each participant in the multilevel regression model captures unobserved characteristics, further reducing potential bias. However, since this study only analyzes the perceived walkability of a small number of young adults aged 19 to 30, further studies are needed to generalize the findings through experiments with a more diverse participant pool, as described in the conclusion (see Lines 574-579).

**Ln 187-197
Subsection Measurement
The content discussed in this subsection pertains to the concept of walkability in your study. Therefore, the description here should be described as a theoretical framework within or after the introduction, not as a material and method. Furthermore, I am concerned that your concept of walkability relies on Alfonz (2005). You should explain the validity of the three dimensions you have set by referring to more studies on walkability.**

- We appreciate your comment. As suggested, we have separated the literature review from the introduction. In the literature review section, we presented a more comprehensive approach to measuring walkability by integrating Alfonzo’s framework with insights from other recent relevant studies to support the dimensions of walkability used in this study (see Lines 129-147).
- Based on the literature review, we selected five dimensions of perceived walkability—safety (for walking and crossing), convenience, attractiveness, and willingness to walk—which together reflect a more holistic understanding of walkability across different street types in the context of Korea. We provided a more detailed explanation and justification for the validity of the selected dimensions of perceived walkability based on previous studies (see Lines 270-290).

**Ln 199-200
Among the five walking needs described above, we employed safety, comfort, and pleasurability.
This terminology of comfort and pleasurability differs from that of Figure 3-1. Is the comfort same as convenience? Is the pleasurability same as attractiveness?**

- We apologize for the confusion. Comfort and pleasurability are concepts used in Alfonzo's framework of walking needs. In this revision, we have comprehensively described how these concepts relate to the convenience and attractiveness used in this study in the literature review (see Lines 138-143). Additionally, in the methodology section, we explained our final selection of comfort and convenience, considering previous research and the context of Korea (see Lines 278-285). In the revised manuscript, we focused solely on the concepts of convenience and attractiveness in the analysis to ensure that readers can understand without confusion.

**Ln 204-205
the willingness to walk on a given road is added as a proxy of overall perceived walkability, consistent with relevant studies[23,24].
I disagree with the idea of measuring walkability by willingness to walk alone. You should make a subsection of the theoretical framework to reinforce the logic.**

- We appreciate your comment. As suggested, willingness to walk alone cannot be considered a proxy for overall perceived walkability. Therefore, in this revision, we have included willingness to walk as one of the five dimensions of perceived walkability, with overall perceived walkability measured using the average of all five dimensions (safety for walking, safety for crossing, convenience, attractiveness, and willingness to walk). A detailed explanation of these methodological approaches and justifications is provided in the measurement section (see Lines 270-290).

**Ln 205-207
For these assessments, we employed a Likert scale ranging from 1 (lowest) to 5 (highest) in response to the question, ‘How do you evaluate the real-time scene?’ for each given scenario.
The results in Figure 3 show that the scores for willingness to walk and attractiveness are mostly similar. I am aware of the problematic questions in your study. Please describe how you asked all the questions, including the safety and comfort questions.**

- As advised, we added specific information on how we asked participants questions about the different dimensions of perceived walkability. A detailed explanation is provided in the Measurement section (see Lines 291–296).
- In addition, the survey questionnaire is included as a supplementary document (S1questionnaire). The five dimensions of perceived walkability are assessed for each scenario (a total of 10 times) immediately after the participant completes the assigned VR experience. For example, we asked: 'This street is safe for walking' using the following questions. (see Supplementary document 1)
- This street is safe for walking.
- This street is safe for crossing
- This street is convenient for walking
- This street is attractive for walking
- This street is the street I want to walk.
- In this revision, we conducted a correlation analysis for all dimensions of perceived walkability to explore the connections among the different types of perceived walkability. Overall, there is a high correlation among the individual variables, but they show differences at that level. The results and findings of this analysis have been added to the Results section (see Lines 340-371).

**Result
Ln 226-227
As shown in Fig 3, we examined five aspects of perceived walkability in our research across different road scenarios.
Wouldn't it be appropriate to analyze Figure 3 from Scenario 1 to Scenario 10, respectively? I cannot understand why the forward and backward are combined in each of the scenarios in Table 1.**

- We apologize for the confusion. One of the initial and main hypotheses of this study was that the vehicle approach directions influence the perceived walkability of one-way roads, particularly in terms of safety. However, the results of the evaluation of perceived safety in each direction did not show any significant differences on the one-way roads, as shown in Fig. 4-1 (Fig. 3-2 in the original manuscript). Since no statistical differences in perceived safety were found based on the direction of approaching vehicles, the comparison of perceived walkability across road types was conducted by aggregating the values from both directions, as shown in Fig. 4-2 (Fig. 3-1 in the original manuscript). To ensure that readers can understand these results without confusion, we added detailed explanations of the process, changed the order of the figures, and provided the results and findings based on this order. (see Lines 373-397)

**Ln 227-229
Panel (1) of Fig 3 displayed five dimensions of perceived walkability across road types, which were statistically significant at least at the 5% level of significance based on the ANOVA test.
Figure 3 should show the significant difference marks between the groups that were significantly different.**

- As suggested, we added the significant difference markers in Fig. 4-2 (originally Fig. 3-1 in the manuscript).

**Ln 259-260**
**The results of the post-experiment survey support the importance of sidewalk presence for safety and walkability.
The results in Figure 3 show that the scores for willingness to walk and attractiveness are mostly similar. Looking at these results, is walkability not more important for attractiveness than safety? The reasons why we should focus on safety are not fully explained.**

- As noted in the previous response to the comment on Lines 205-207, we conducted a correlation analysis for all dimensions of perceived walkability to explore the connections among the different types of perceived walkability. Overall, there is a high correlation among the individual variables, but they show differences at that level. The results and findings of this analysis have been added to the Results section (see Lines 340-371).
- Safety for crossing shows a relatively lower correlation with the other dimensions, implying that policy efforts to improve this aspect should be emphasized as suggested by this comment. We noted this as follows.

“*The correlation-based finding could suggest priorities for designing effective interventions for urban walkability enhancement. Improvements in high correlation pairs may simultaneously elevate overall walkability, while low correlation ones require targeted interventions with more effort to ensure both dimensions reach optimal levels* (see Lines 367-370).”

**Ln 310-323
For all five dimensions of perceived walkability --- more dedicated knowledge of walkable cognition and behaviors.
I think this is the main part of your results. You should be a little more specific, describing your scores, including odds ratios. Another important result is that age is significantly related in the control group.**

- As suggested, we added a specific description of the odds ratios shown in Table 3 to present the variation of perceived walkability across street types. In general, the results represent the expected pattern: pedestrian-only streets, wide sidewalks, narrow sidewalks, and shared streets for most dimensions, except for safety for crossing. The interpretation of these results is provided. (see Lines 471-487).
- We appreciate your detailed comment on the effect of Age. We added the description of the results (see Lines 503-509).

**Discussion
The point you are missing in your discussion is the relationship between the elements of walkability. Your study was analyzed in terms of safety, comfort, attractiveness, and willingness to walk. However, with respect to the results, you focus too much on safety. You must add a paragraph on the relationship between the elements, based on the theoretical frame of reference.**

- As suggested, we conducted a correlation analysis for all dimensions of perceived walkability to explore the connections among the different types of perceived walkability. Overall, there is a high correlation among the individual variables, but they show differences at that level. The results and findings of this analysis have been added to the Results section (see Lines 340-371 and Fig. 3).
- In addition, we tried to have comprehensively discussed the analysis results of various dimensions of walkability, not only safety, throughout the revised manuscript and presented their implications.
